# The effects of caffeine in adults with neurogenic orthostatic hypotension: a systematic review

**DOI:** 10.1007/s10286-021-00814-5

**Published:** 2021-06-18

**Authors:** Jake Ryan Gibbon, James Frith

**Affiliations:** Population Health Sciences Institute, Newcastle University, Newcastle Upon Tyne, UK

**Keywords:** Neurogenic orthostatic hypotension, Postural hypotension, Caffeine

## Abstract

**Purpose:**

To systematically review the evidence base for the effectiveness and safety of caffeine for the treatment of neurogenic orthostatic hypotension in adults.

**Methods:**

Eight electronic databases were searched in January 2021. Original research studies or case reports involving adults with neurogenic orthostatic hypotension were included if caffeine was an intervention and outcomes included symptoms, blood pressure or adverse effects. Relevant studies were screened and underwent qualitative analysis. Insufficient reporting precluded meta-analysis.

**Results:**

Five studies were identified: four crossover studies and one case report summation. Study size ranged from 5 to 16 participants. Participants had neurogenic orthostatic hypotension, with a mean standing systolic blood pressure of 86 mmHg. Two studies evaluated caffeine alone. Three studies administered caffeine in combination with ergotamine. Caffeine dose ranged from 100 to 300 mg. Nature and timing of outcomes measured varied between studies, with measurements being recorded from 30 to 480 min after intervention. Caffeine/ergotamine improved symptoms in one study and reduced orthostatic blood pressure drop in two studies. Caffeine/ergotamine increased seated blood pressure in three studies, whilst the results for caffeine alone were inconsistent. No serious adverse events were reported. All studies demonstrated high risk of bias.

**Conclusion:**

Caffeine should only be considered as a treatment for adults with neurogenic orthostatic hypotension when evidence-based treatments have been exhausted.

**Systematic review registration:**

PROSPERO ID: CRD42020124589. Date of registration: 30/10/2020

**Supplementary Information:**

The online version contains supplementary material available at 10.1007/s10286-021-00814-5.

## Introduction

Orthostatic hypotension (OH) is defined as a sustained drop in blood pressure (BP, ≥ 20 mmHg systolic BP or ≥ 10 mmHg diastolic BP) within 3 min of standing upright [1]. It increases the risk of falls and all-cause mortality and is associated with disabling symptoms [2, 3]. It is very common, especially in older people and in those with chronic disease [4].

Neurogenic OH (nOH), a pathophysiological subtype of OH, results from central or peripheral autonomic dysfunction, leading to impairment of baroreflex-mediated vasoconstriction of skeletal muscle and splanchnic vasculature [5]. Both pharmacological and non-pharmacological treatments for nOH are poorly evidenced, and issues with efficacy, adherence and tolerability are common, creating a need for a better-quality evidence base [3].

Caffeine is a widely available, inexpensive food constituent with few side effects or associations with poor health outcomes [6, 7]. It has vasoconstrictive properties through antagonism of adenosine receptors (A1, A2A and A2B) [8] and has been shown to modestly increase BP both acutely and in the longer term in healthy individuals [9]. Low-quality evidence indicates that caffeine is an effective treatment for postprandial hypotension, another common problem in patients with autonomic failure, potentially through inhibition of adenosine-driven splanchnic vasodilatation [10]. This has led to the hypothesis that caffeine may be helpful for people with nOH. Indeed, caffeine has consequently been recommended as a treatment for refractory nOH in the literature and in clinical practice, although there is no consensus regarding its efficacy [10, 11].

Due to the significant uncertainty regarding the benefit of caffeine in nOH, a systematic review of its efficacy and safety was undertaken, evaluating the effectiveness and safety of caffeine on OH in adults, focussing on caffeine’s effects on BP, symptoms and adverse events.

## Methods

### Criteria for considering studies for this review

#### Participants

Adults (aged over 18 years) diagnosed with OH, as defined by the international consensus criteria in 1996 [12] or the 2011 update [1]. If diagnostic criteria were not stated, the reviewers must have been able to determine that the participants met the diagnostic criteria from the blood pressure data presented. Any underlying cause of OH was eligible for inclusion. Participants could be based in any setting (e.g. community, hospital, nursing home).

#### Intervention

Caffeine administered orally in any form, dose or duration. Presence of a control or comparator group was not required due to the anticipated lack of studies.

#### Outcomes

Studies were considered if the outcomes measured included any of the following: symptoms, diagnostic vital sign changes (e.g. orthostatic BP drop), change in resting BP or adverse effects/events.

#### Study type

A wide range of study types were considered in order to have a sensitive search strategy, as it was anticipated there would be a limited number of research studies on this topic. Original research studies including randomised control trials, crossover studies, observational studies and case series were eligible.

### Search methods for identification of studies

Scoping work during an initial search of MEDLINE and the Centre for Reviews database (https://www.crd.york.ac.uk/CRDWeb/) was undertaken to identify keywords and terms from previous studies and review articles, to inform the search strategy. Because the scoping work did not reveal a high number of results, search terms specific to clinical trial type were not used.

Published articles were searched for using MEDLINE (1946 to week 2 January 2019), EMBASE (1974 to 22 January 2019), PubMed (no date limits) and Scopus (no date limits). Conference proceedings and theses were identified using Web of Science (1970–2019) and ProQuest (1970–2019). Grey literature was sought using Open Grey (no date limits). Ongoing or unpublished studies were searched for using the World Health Organization International Clinical Trials Registry Platform. Reviewers also searched reference lists when reviewing full-text articles. Searches were performed in January 2019. A list of search terms for each database is included in the Supplementary file.

Due to the COVID-19 pandemic, the study was paused in 2020, creating a gap between the search date and publication date. For this reason, the database search was repeated in January 2021 using the same search strategy but restricted to the dates January 2019 to January 2021. One hundred and four additional studies were identified from this update, which were all excluded in primary screening (title only).

### Data collection and analysis

#### Selection of studies

All identified studies were collated into Endnote X9, where duplicates were removed. Primary screening was then carried out (title only), followed by secondary screening (abstract). All potentially eligible studies progressed to review of the full text to assess eligibility. All eligibility assessments were carried out by two reviewers (JG and JF).

#### Data extraction and management

Data were extracted from all included studies by JG and verified by JF, using forms based on the Cochrane Collaboration’s Data Collection Form for Intervention Reviews [13]. This included study design; methodology; participant characteristics; intervention nature, dosage, form of administration and duration; funding and duration of study and study outcomes. In addition to the outcomes required for inclusion, the following outcomes were extracted if they were available: activities of daily living, change in resting BP, adherence to treatment regime and orthostatic tolerance (time to onset of symptoms during upright posture).

At each stage, an independent arbitrator was available if the two reviewers disagreed; however, this was not required.

#### Assessment of risk of bias in included studies

The quality of the included studies was assessed independently by JG and JF. Criteria described in the Cochrane Handbook for Systematic Reviews of Interventions [13] were followed, consisting of risk of bias from selection, performance, detection, attrition, reporting and any additional bias identified. Risk of bias in each area was judged as high, low or unclear.

#### Data synthesis and analysis

Due to high heterogeneity in the data and incompletely reported outcomes, meta-analysis of data was not possible for any of the outcomes.

#### Protocol and registration

The review protocol was registered prospectively (accessed at: crd.york.ac.uk/PROSPERO/ ID: CRD42020124589). Changes to the planned protocol: Originally the study intended to study the effect of caffeine on orthostatic intolerance, including OH, postural tachycardia syndrome (PoTS) and neurally mediated syncope. However, initial scoping work revealed no relevant studies for PoTS, neurally medicated syncope or OH of non-neurogenic aetiology. Therefore, the protocol was adapted to focus solely on nOH.

## Results

### Study selection

**Fig. 1:**
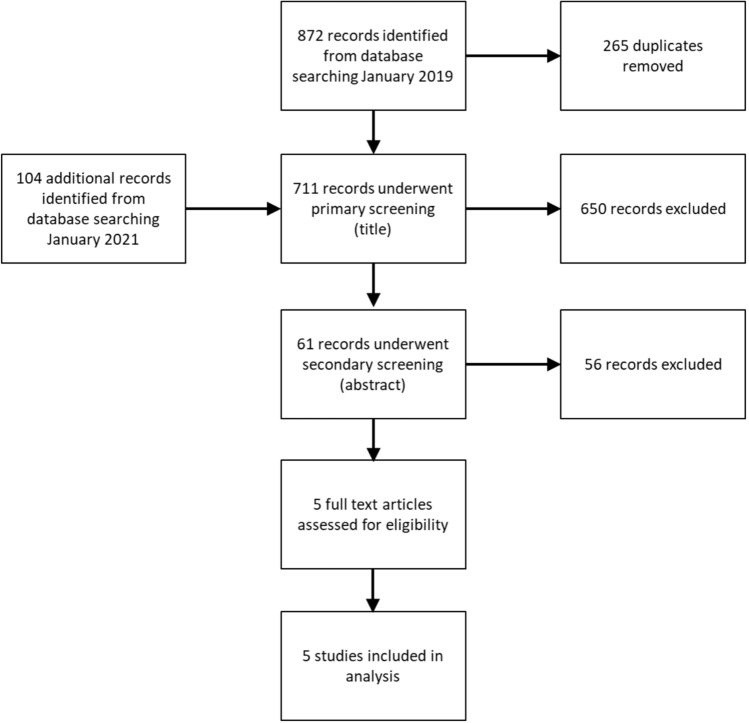
Study selection process

The study selection process is summarised in Fig. 1.

### Description of studies

All five included studies were based in the United States, were single-centre and were reported in English. The number of participants included in the studies ranged from 5 to 16, the mean age of participants ranged from 64 to 69 years, and all participants had neurogenic OH, which was predominantly due to Parkinson’s disease (PD), multisystem atrophy (MSA) and pure autonomic failure (PAF).

One study was a summation of case reports, and four studies were crossover trials, with participants receiving caffeine and placebo at different time points. The duration of the crossover studies ranged from 2 to 7 h, involving a single dose of oral caffeine, administered in tablet form.

The characteristics of the five studies meeting the inclusion criteria are summarised in Table 1.

**Table 1:** Characteristics of included studies

Arnold et al. [17]	
Methods	Design: crossover study
	Allocation: randomised, computerised
	Blinding: single blind, participants were blinded
	Duration: 100 min
	Setting: single location, Vanderbilt General Clinical Research Center, Nashville, TN, USA
Participants	Diagnosis: neurogenic (‘severe autonomic impairment ‘) OH (international consensus criteria)
	Aetiology: PAF (*n* = 8), MSA (*n* = 2) and PD (*n* = 2)
	*N*: entered: 12 randomised: 12 completed. Outcomes available in 5–12, dependant on outcome
	Female: 66.6%
	Age: 64.2 (9.80) years
	Severity: mean standing SBP: 80 (16.9) mmHg
	Mean orthostatic change in BP: 56 (20.2) mmHg
Interventions	A single dose of the following interventions was administered with around 50 ml water, at least 2 h after a meal:
	Combination 1 mg ergotamine and 100 mg caffeine tablet (Cafergot, Novartis Pharmaceuticals)
	Midodrine, 5 or 10 mg. Participants given dose of midodrine they were prescribed to take regularly. Mean dose 8.33 (2.46) mg
	Placebo—nature unspecified
Outcomes	Primary: change in seated SBP during the 60 min post-drug period compared to 30 min pre-drug administration
	Secondary: Orthostatic tolerance at baseline and 60 min post-drug administration; difference in overall symptoms and in light-headedness (measured using the OHQ [20] from baseline to 60 min post-drug administration; number of patients able to stand for 10 min, at 60 min post-drug administration; number of patients with an increase in systolic seated BP of ≥ 20 mmHg at 60 min post-drug administration from baseline
Other	Data presented as: mean (95% confidence interval)
Jordan et al. [18]	
Methods	Design: crossover study
	Allocation: non-randomised, based on ‘intentions for long term therapy’
	Blinding: single blind, no further description
	Duration: 120 min
	Setting: single location, Elliot V. Newman Clinical Research Center at Vanderbilt University Medical Center, USA
Participants	Diagnosis: OH (international consensus criteria)
	Aetiology: MSA (*n* = 20) or PAF (*n* = 15)
	*N*: entered: unclear, completed: 35, 16 allocated to caffeine
	Female: 31%
	Age: 67 ± 2 years
	Severity: orthostatic systolic BP drop: MSA: −63 ± 6.5 mmHg, PAF: −69 ± 4.5 mmHg
Interventions	A single dose of one of the following interventions was administered with 50 ml water after being seated for 30 min and at least 2.5 h after breakfast or lunch
	Oral phenylpropanolamine tablet 12.5 mg
	Oral yohimbine tablet 5.4 mg
	Oral indomethacin 50 mg
	Oral ibuprofen tablet 600 mg
	Oral caffeine tablet 250 mg
	Oral methylphenidate tablet 12.5 mg
	Oral placebo tablet (lactose, Spectrum, Gardena, CA, USA)
	Oral phenylpropanolamine tablet 25 mg
	Oral midodrine tablet 5 mg
Outcomes	Outcomes not specified prior to the results section
	Seated SBP change 0–120 min after intervention compared to ‘baseline’, determined by averaging five consecutive SBP readings taken prior to administration of intervention
	Peak seated SBP 0–120 min after intervention
	Time to peak seated SBP 0–120 min after intervention
	Proportion of participants that ‘responded’ to the interventions, defined as AUC_drug_ − AUC_placebo_ >0 mm/min, where AUC refers to seated SBP on the *y*-axis and time on the *x*-axis. Values calculated between 30 and 120 min after intervention administration
Other	Data presented as: mean (standard error of the mean)
Dewey et al. [18]	
Methods	Design: Open-label trial/case series
	Allocation: all patients received experimental drug
	Blinding: none
	Duration: effect of single dose testing: 120 min. Otherwise: 1 week to 14 months
	Setting: single location, University of Texas Southwestern Medical Center, Dallas, TX, USA
Participants	Diagnosis: OH (international consensus criteria as per baseline data). OH (fulfil international consensus criteria per baseline data provided)
	Aetiology: PD (*n* = 2) or MSA (*n* = 6)
	*N*: entered: unclear, completed: 8
	Female: 50%
	Mean age: 69 years
	Severity: no objective measure, ‘Previously failed treatment with the usual physical measures (dietary salt supplementation, support hose, elevation of head of bed) and fludrocortisone’
Interventions	Ergotamine 1 mg/caffeine 100 mg tablet. 1–3 tablets were administered for one-off dose; 1–5 tablets/day for regular treatment. Reason for dosing variation not elucidated
Outcomes	Outcomes not specified prior to the results section
	Effect of single dose of ergotamine caffeine on standing SBP (measured at 0 and 75–120 min after intervention)
	Mean supine/sitting and standing MAP before and during treatment with ergotamine/caffeine (time point not specified)
	Symptomatic response (defined as transient or persistent reduction of symptoms) and adverse effects to ergotamine/caffeine after 1 week–14 months of intervention
Hoeldtke et al. [15]	
Methods	Design: crossover study
	Allocation: ‘Random sequence’. Randomisation method not specified
	Blinding: not specified
	Duration: 7 h
	Setting: single location (General Clinical Research Center of Temple University Hospital, Philadelphia, PA, USA)
Participants	Diagnosis: four patients had OH (international consensus criteria as per baseline data). One patient had ‘postprandial hypotension’ (criteria for diagnosis not defined) due to ‘alcoholism’
	Aetiology: OH due to diabetes, alcoholism or idiopathic.
	*N*: entered: unclear; randomised: unclear; completed: 5
	Female: unclear
	Age: unclear
	Severity: unclear
Interventions	A single dose of the following interventions was given on four consecutive days:
	Dihydroergotamine (10 µg/kg) subcutaneous injection. Given at 07:00. Dilutant not stated
	Caffeine tablet (250 mg). Given at 07:30. Volume of liquid used to swallow pill not mentioned
	Dihydroergotamine (administered as above) plus caffeine (administered as above)
	‘Placebo injection’ (0.9% sodium chloride solution). Volume and time given not specified
Outcomes	Outcomes not specified prior to the results section
	Effect on seated MAP 0–480 min after intervention compared to placebo
	Adverse effects after 1–4 months of intervention
Onrot et al. [16]	
Methods	Design: crossover study
	Allocation: randomised, method not defined
	Blinding: not specified
	Duration: dependent on trial
	Setting: single location, Elliot V. Newman Clinical Research Center of Vanderbilt University, TN, USA
Participants	Diagnosis: OH (international consensus criteria as per baseline data)
	Aetiology: PAF, MSA
	*N*: entered: unclear; randomised: unclear; completed: 5
	Female: 40%
	Age: 64 (5.87) years
	Severity: mean standing SBP: 91 (20.8) mmHg
	Mean orthostatic change in BP: 62 (23.2) mmHg
Interventions	For at least 3 days before the trial period, patients abstained from methylxanthine-containing beverages and all medications. Patients were also placed on a diet containing 150 mmol of sodium and 80 mmol of potassium
	Patient were seated after an overnight fast during all interventions.
	Caffeine 250 mg capsule with 100 ml of water 30 min before a standardised meal, single dose
	Placebo (form unclear) 30 min before standardised meal, single dose
Outcomes	Outcomes not specified prior to the results section
	Seated blood pressure and heart rate change 0–120 min after intervention
Other	Data presented as mean (standard deviation)

*BP* blood pressure, *MAP* mean arterial pressure, *MSA* multisystem atrophy, *OH* orthostatic hypotension, *OHQ* orthostatic hypotension questionnaire, *PAF* pure autonomic failure, *PD* Parkinson’s disease

### Participants

Participants in all five studies fulfilled international consensus criteria for OH. Three studies [14–16] did not state the diagnostic criteria used, but we were able to confirm OH from baseline data presented in each study.

Regarding OH severity, the mean standing BP was 86 mmHg and mean postural change in systolic blood pressure was 58.8 mmHg amongst participants from two studies (*n* = 24) [16, 17].

A total of 46 participants who received caffeine remained at completion. Recruitment and withdrawal data for the studies were not available.

Mean age of participants cannot be calculated with the available data, as two studies did not clarify the age of the participants involved in the arm of the studies that involved caffeine administration [15, 18].

### Interventions

Caffeine was administered orally in tablet form in all five studies. In three studies the caffeine was administered in combination with ergotamine. Ergotamine is a multimodal vasoconstrictor, and caffeine has been shown to increase ergotamine’s intestinal absorption [19]. This took the form of a combination tablet in two studies [14, 17] and subcutaneous injection 30 min prior to caffeine administration in one study [15]. The caffeine dose administered ranged from 100 to 300 mg, and the mean was 189.1 ± 75.9 mg.

### Methods

Three studies were randomised [15–17], although in two of these the method of randomisation was not specified [15, 16]. Two studies were single-blinded (participants) [17, 18]. One study was non-blinded [14], and the blinding of the remaining studies is unclear [15, 16]. All four crossover studies involved a single dose of intervention, with physiological responses being measured up to 60 min to 8 h after administration.

### Effects of interventions

Findings of studies are summarised in Table 2.

**Table 2:** Summary of findings

Symptoms	
Arnold et al. [17]	Ergotamine/caffeine significantly reduced overall symptoms (*p* = 0.034) and light-headedness (*p* = 0.040). The size of the effect is unclear
Dewey et al. [14]	‘Transient or persistent reduction of symptoms during outpatient use of the drug’ occurred in six out of eight participants given ergotamine/caffeine treatment in the longer term (time not specified)
Change in orthostatic blood pressure drop	
Dewey et al. [14]	Pre-post comparison of ergotamine/caffeine, orthostatic SBP drop reduced by 16.50 (± 10.11) mmHg and DBP by 11.33 (± 9.91) mmHg at 75–120 min In the longer term (time not specified), caffeine/ergotamine treatment resulted in a reduction in orthostatic SBP drop of 44.25 (± 31.05) mmHg and DBP by 5.83 (± 19.76) mmHg, compared to pretreatment
Adverse events	
Hoeldtke et al. [15]	One out of 12 participants experienced heartburn after a single dose of a caffeine tablet
Dewey et al. [14]	Three out of eight participants stopped taking ergotamine/caffeine due to side effects (nausea, atypical chest pain and supine hypertension)
Arnold et al. [17]	One of the five participants who continued ergotamine/caffeine post-study stopped taking the medication due to ‘feeling tense’
Seated blood pressure	
Arnold et al. [17]	Ergotamine/caffeine significantly increased seated SBP compared to placebo (slope difference: 1.003; 95% CI 1.001–1.005; *p* = 0.003) but not when compared to midodrine (slope difference: 1.000 95% CI 0.998–1.001; *p* = 0.621). Nine out of 12 participants’ seated SBP increased by ≥ 20 mmHg with ergotamine/caffeine, compared to 5 out of 12 with midodrine; the difference was non-significant (*p* = 0.125)
Dewey et al. [14]	Ergotamine/caffeine increased supine/seated BP, with SBP rising by 23.63 (± 16.76) mmHg and DBP rising by 16 (± 17.77) mmHg, 75–120 min after administration. In the longer term (time not specified), seated SBP decreased by 2.13 (± 30.21) mmHg, and DBP increased by 1.75 (± 12.41) mmHg when compared to pretreatment
Hoeldtke et al. [15]	Ergotamine/caffeine treatment increased the area under the curve for MAP in five patients with OH more effectively than ergotamine or caffeine monotherapy or placebo from baseline to 480 min after administration (*p* < 0.05). Graphical data appears to show that caffeine monotherapy increases seated MAP compared to placebo consistently from 0 to 480 min after administration, but significance testing for this was not carried out
Onrot et al. [16]	Caffeine monotherapy led to an initial rise in seated BP, from 129 ± 25/78 ± 12 at baseline to 141 ± 30/84 ± 16 mmHg after 45 min (*p* < 0.01). The effect on SBP became non-significant between 75 and 90 min, whilst the effect on DBP remained significant up to 120 min. MAP 1 h after caffeine ingestion was significantly higher (*p* < 0.05) than ‘before’ caffeine ingestion; no data are provided to confirm this
Jordan et al. [18]	No significant difference in peak seated SBP in the 120 min after administration of caffeine compared to baseline SBP or peak SBP after placebo administration in the same time period
Standing blood pressure	
Dewey et al. [14]	Ergotamine/caffeine increased SBP by 39.83 (± 10.40) mmHg and DBP by 17.16 (± 9.17) mmHg, 75–120 min after administration
	In the longer term, standing SBP increased by 42.13 (± 21.05) mmHg, and DBP rose by 8.33 (± 15.19) mmHg (time point not specified) when compared to pretreatment
Orthostatic tolerance	
Arnold et al. [17]	Area under the curve for postural SBP during 10 min of standing was not statistically different between ergotamine/caffeine, midodrine or placebo
	The percentage of participants able to stand for 10 min, 60 min after administration of ergotamine/caffeine, midodrine or placebo was not significantly different: 66.6, 50 and 41.7%, respectively

*BP* blood pressure, *DBP* diastolic blood pressure, *MAP* mean arterial pressure, *SBP* systolic blood pressure

### Symptoms

Symptoms were reported in two studies. In Arnold’s paper [17], ergotamine/caffeine significantly reduced overall symptom severity, measured using the Orthostatic Hypotension Questionnaire’s (OHQ) [20] composite score (*p* = 0.034) and light-headedness component (*p* = 0.040) at 60 min. In contrast, there was no significant effect on symptoms with midodrine or placebo. However, the size of the effect is unclear, and each arm of the study was not compared directly.

Dewey [14] also reported symptom improvement, defined as a ‘transient or persistent reduction of symptoms during outpatient use of the drug’, in six out of eight patients who were administered ergotamine/caffeine treatment. However, the time point at which this was assessed is unclear, and there is no evidence that their method of measuring symptom burden had been validated.

Data regarding symptomatic response to caffeine as a monotherapy was not collected in any of the studies reviewed.

### Orthostatic BP drop

In one study [14], when comparing baseline to post-ergotamine/caffeine (measured at 75–120 min), orthostatic SBP drop was reduced by 16.50 (± 10.11) mmHg and DBP drop was reduced by 11.33 (± 9.91) mmHg. When comparing baseline to ‘during therapy’ (unclear time point), caffeine/ergotamine treatment resulted in a reduction in orthostatic SBP drop of 44.25 (± 31.05) mmHg and DBP by 5.83 (± 19.76) mmHg.

Arnold [17] measured postural SBP 60 min post-ergotamine/caffeine, at baseline (seated) and after 1, 3, 5 and 10 min of standing. The area under the curve (AUC) was calculated and compared to placebo and midodrine. No statistical difference was found between ergotamine/caffeine and midodrine (ΔAUC_SBP_: −163; 95% CI −387 to 62; *p* = 0.155) or ergotamine/caffeine and placebo (ΔAUC_SBP_: 248; 95% CI −73 to 568; *p* = 0.130).

### Change in standing blood pressure

Summation of individual participant data from Dewey’s study [14] reveals that a single dose of ergotamine/caffeine increased standing BP, with SBP rising by 40 (± 10.40) mmHg and DBP rising by 17 (± 9.17) mmHg 75–120 min after administration.

Standing SBP also increased by 42.13 (± 21.05) mmHg and DBP rose by 8.33 (± 15.19) mmHg ‘during therapy’ (time point not specified) when compared to pretreatment.

### Change in seated blood pressure

Arnold’s [17] study compared the effect of ergotamine/caffeine, placebo and midodrine on seated SBP, measuring seated SBP 30 min before and 60 min post-intervention.

Ergotamine/caffeine significantly increased seated SBP compared to placebo (slope difference: 1.003; 95% CI 1.001–1.005; *p* = 0.003). However, in comparison to midodrine, there was no significant difference (slope difference: 1.000 95% CI 0.998–1.001; *p* = 0.621). Nine out of 12 participants’ seated SBP increased by  ≥ 20 mmHg with ergotamine/caffeine, compared to 5 out of 12 with midodrine, although the difference in findings was non-significant (*p* = 0.125), and the effect of placebo was not reported.

Summation of individual participant data from Dewey’s study [14] demonstrated that a single dose of ergotamine/caffeine increased supine/seated BP, with SBP rising by 24 (± 16.76) mmHg and DBP rising by 16 (± 17.77) mmHg on average, 75–120 min after administration. However, this effect was not seen in the longer term, with seated SBP falling by 2 (± 30.21) mmHg and DBP rising by 2 (± 12.41) mmHg ‘during therapy’ (time point not specified) when compared to pretreatment.

Hoeldtke [15] found that ergotamine/caffeine treatment increased mean arterial pressure (MAP) in five patients with OH more effectively than ergotamine or caffeine monotherapy or placebo when areas under the curve from baseline to 480 min after administration were compared (effect size not reported, *p* < 0.05). Graphical data from Hoeltdke’s study [15] also appears to show that caffeine monotherapy increases seated MAP compared to placebo consistently from 0 to 480 min after administration, but significance statistical testing for this was not carried out.

Onrot [16] demonstrated that administration of caffeine monotherapy lead to an initial significant rise in seated BP, from 129 ± 25/78 ± 12 at baseline to 141 ± 30/84 ± 16 mmHg after 45 min (*p* < 0.01). The effect on systolic blood pressure became non-significant between 75 and 90 min post-caffeine ingestion, whilst the effect of diastolic blood pressure remained significant up to 120 min (end of observation period). They also report that mean arterial pressure 1 h after caffeine ingestion was significantly higher (*p* < 0.05) than ‘before’ caffeine ingestion, but no data are provided to confirm this.

Jordan [18] found no significant difference in the peak seated SBP in the 120 min after administration of caffeine compared to baseline SBP or peak SBP after placebo administration within the same time period.

### Orthostatic tolerance

Arnold’s study [17] reported the percentage of participants able to stand for 10 min, 1 h after administration of ergotamine/caffeine, midodrine or placebo. The result was not significantly different, with 66.6, 50 and 41.7% of participants in each arm able to stand for 10 min, respectively.

### Other outcomes

Activities of daily living and adherence to therapy (in longer-term studies) were not reported in any of the identified studies.

### Adverse events

The frequency and nature of adverse events were inadequately recorded (non-systematically or not at all). Hoeldtke [15] reported side effects in one out of 12 participants after single administration of caffeine tablet (heartburn). Dewey [14] reported that three out of eight participants stopped taking ergotamine/caffeine due to side effects: after 1 week due to nausea, 2 weeks due to ‘atypical chest pain’ and 14 weeks due to supine hypertension. In Arnold’s study [17], one out of five patients who continued ergotamine/caffeine after the study stopped taking the medication after an unspecified duration of time due to ‘feeling tense’. Two studies did not report adverse events [16, 18].

### Risk of bias across studies

Risk of bias is summarised in Table 3. Overall, all studies were of a high risk of bias. As fewer than 10 studies were included, a funnel plot of reporting bias was precluded [13].

**Table 3:** Risk of bias

	Randomisation	Allocation	Participant and personnel blinding	Blinding of outcome assessment	Attrition bias	Reporting bias	Other bias
Arnold [17]	Low	Unclear^c^	High^g^	Unclear^j^	Unclear^k^	High^lmn^	Low
Dewey et al. [14]	High^a^	High^d^	High^h^	Unclear^j^	Unclear^k^	High^lno^	High^p^
Hoeldtke et al. [15]	Unclear^b^	High^e^	Unclear^i^	Unclear^j^	Unclear^k^	High^lno^	High^q^
Jordan et al. [18]	High^a^	High^f^	High^g^	Unclear^j^	Unclear^k^	High^lno^	Low
Onrot et al. [16]	Unclear^b^	High^e^	Unclear^i^	Unclear^j^	Unclear^k^	High^lno^	Low

^a^No randomisation

^b^Method of randomisation not specified

^c^Method of allocation not specified

^d^No allocation occurred

^e^Variation in route of administration between interventions

^f^Allocation of medication based on intention for long-term therapy, therefore could be predicted

^g^Single-blinded

^h^Unblinded

^i^Blinding not specified

^j^Blinding of outcome assessors not specified

^k^Recruitment and withdrawal not specified

^l^Author-derived outcome measures

^m^Outcomes missing from results section

^n^Raw data not provided for graphical figures

^o^No outcomes specified prior to results section

^p^Method of significance testing not mentioned. Study sponsors not mentioned

^q^Study sponsor was the drug manufacturer

## Discussion

This systematic review has found a lack of good-quality evidence for the use of caffeine in nOH. The studies reviewed highlight that caffeine, particularly when in combination with ergotamine, may cause short-term improvements in blood pressure and symptom burden in patients with nOH, but due to the poor quality of evidence, caffeine can only be recommended when other evidence-based treatment options have been exhausted. As no studies were identified involving participants with non-neurogenic OH, no conclusions can be drawn about caffeine’s effects in this patient group.

All included studies were small and took place in one of three sub-specialised centres. Studies were limited to participants with the alpha-synucleinopathies PD, MSA or PAF. Participants tended to be around retirement age and, based on the data presented, appear to have had relatively severe nOH. With little to no information provided about participant comorbidity, concurrent medication or performance status, it is difficult to judge how representative these participants are and whether they reflect the usual clinical patient with nOH.

In the reviewed studies, caffeine was administered as an oral tablet, either as a monotherapy or in combination with ergotamine, at a dose of 100–250 mg. There were no identified studies evaluating the effect of caffeine on OH in other preparations, such as within widely consumed hot beverages like tea or coffee.

All of the studies included in this review were found to be of high risk of bias in multiple domains, potentially a reflection of a lack of formal research reporting guidelines at the time the studies were conducted (four out of five were published between 1986 and 1998). Trial methodology, including identification, selection, randomisation, allocation and blinding, was poorly performed or poorly described. In general, inclusion and exclusion criteria and participant selection were poorly described, increasing the risk of selection bias. Attrition was also poorly addressed in all included studies. The risk of reporting bias was high in all papers. A lack of trial registration, author-derived outcomes and missing data were common, leading to potential publication bias. Furthermore, in four studies no outcome measures were provided in the methodology section [14–16, 18]. There were potential conflicts of interest in two studies, with one study being sponsored by the experimental drug manufacturer [15] and in the other, uncertainty over who the study sponsor was [14].

Pooled quantitative analyses could not be performed on any of the outcomes due to significant heterogeneity in the outcomes measured and incomplete reporting of data. Although all studies measured the effect of caffeine or ergotamine/caffeine on participant’s seated/supine BP, the data in four out of five papers were only displayed graphically. Only one study measured the clinically important impact on orthostatic blood pressure changes [14] and effect on symptom burden [17], and this was only after a single dose of ergotamine/caffeine. A further limitation to meta-analysis was the varied timing of outcome measurement. Although all included studies measured the very short-term effects of caffeine, the timing varied widely. The short-term nature of the outcome measurement also limits the external validity of the findings, with the effectiveness of caffeine at 1 h post-dose not being particularly clinically useful.

As meta-analysis could not be undertaken, the effect size and variance of the outcomes studied are unclear. The quality of the evidence found in this review is poor, with significant bias, in the studies reviewed. Before ergotamine/caffeine can be considered as a treatment for nOH, further larger-scale, methodologically sound studies are needed to validate the above findings. Such studies should include other clinically important outcomes, such as ability to undertake activities of daily living, falls and adverse events. These studies should also aim to evaluate the effectiveness of caffeine in the long term.

That being said, there are significant barriers to conducting the idealistic studies described above, which may also go some way to explain some of the shortcomings of the studies reviewed in this systematic review.

nOH is a rare disease requiring specialist diagnosis and management [5]. As a consequence, most experimental studies involving nOH are carried out in a limited number of sub-specialist centres with a large enough patient cohort, leading to small sample sizes and selection bias. Conducting longer-term studies into caffeine may also be challenging due to the lack of pharmaceutical funding for such a trial involving a generic drug [21]. Indeed, recent therapeutic advances in nOH have been sponsored by the pharmaceutical industry [22].

There are several limitations to this systematic review. In order to create a sensitive rather than specific search strategy, this review includes non-randomised studies, which will naturally lower the quality of evidence judgements. However, given the lack of studies in this area, this became necessary. Although a wide range of data sources were utilised in the study identification process, it did not include certain regional specific databases or non-English studies. It is possible that pooling of data could have become possible if data were sought from the study authors.

In conclusion, due to lack of good-quality evidence to support or refute its use, caffeine should only be considered as a treatment for adults with nOH when evidence-based treatments have been exhausted.

## Supplementary Information

Below is the link to the electronic supplementary material.

Supplementary file1 (DOCX 30 KB)

## Data Availability

Not applicable.
