# Supplementary material for: The effects of caffeine in adults with neurogenic orthostatic hypotension: a systematic review
Source: Clin Auton Res. 2021 Jun 18;31(4):499–509. doi: 10.1007/s10286-021-00814-5 (PMC8212790; doi:10.1007/s10286-021-00814-5)
Supplement: Supplementary file 1 — Supplementary file1 (DOCX 30 KB) [file 10286_2021_814_MOESM1_ESM.docx]

Supplementary file

# Database search results

| **Database** | **Number of records 2019** | **Additional records 2021** |
| --- | --- | --- |
| Medline | 26 | 1 |
| Embase | 228 | 37 |
| PubMed | 260 | 31 |
| ProQuest | 58 | 1 |
| Scopus | 221 | 24 |
| ISRCTN registry | 5 | 3 |
| Web of Science | 74 | 7 |
| Open Grey | 0 | 0 |

# Data base searches January 2019

### Medline 1946 to week 2 January 2019

Adding search term faintness added no additional end results.

| **#** | **Searches** | **Results** |
| --- | --- | --- |
| 1 | Hypotension, Orthostatic/ | 5431 |
| 2 | orthostatic hypotension.mp. | 4385 |
| 3 | postural hypotension.mp. | 1339 |
| 4 | Orthostatic Intolerance/ | 276 |
| 5 | orthostatic intolerance.mp. | 1130 |
| 6 | neurally mediated syncope.mp. | 405 |
| 7 | Syncope, Vasovagal/ | 1801 |
| 8 | vasovagal syncope.mp. | 1236 |
| 9 | reflex syncope.mp. | 163 |
| 10 | situational syncope.mp. | 30 |
| 11 | neurogenic syncope.mp. | 31 |
| 12 | neurocardiogenic syncope.mp. | 439 |
| 13 | Postural Orthostatic Tachycardia Syndrome/ | 373 |
| 14 | postural orthostatic tachycardia syndrome.mp. | 486 |
| 15 | postural tachycardia syndrome.mp. | 329 |
| 16 | fainting.mp. | 992 |
| 17 | presyncope.mp. | 901 |
| 18 | carotid sinus syncope.mp. | 82 |
| 19 | Carotid sinus syndrome.mp. | 361 |
| 20 | 1 or 2 or 3 or 4 or 5 or 6 or 7 or 8 or 9 or 10 or 11 or 12 or 13 or 14 or 15 or 16 or 17 or 18 or 19 | 13232 |
| 21 | Caffeine.mp. or CAFFEINE/ | 30198 |
| 22 | TEA/ or tea.mp. | 24820 |
| 23 | coffee.mp. or COFFEE/ | 11861 |
| 24 | 21 or 22 or 23 | 61020 |
| 25 | 20 and 24 | 38 |
| 26 | limit 25 to animals | 4 |
| 27 | limit 25 to "review" | 8 |
| 28 | 26 or 27 | 12 |
| 29 | 25 not 28 | 26 |

### Embase (1974 to 2019 January 22)

| **#** | **Searches** | **Results** |
| --- | --- | --- |
| 1 | Hypotension, Orthostatic/ | 13551 |
| 2 | orthostatic hypotension.mp. | 20994 |
| 3 | postural hypotension.mp. | 1871 |
| 4 | Orthostatic Intolerance/ | 985 |
| 5 | orthostatic intolerance.mp. | 2072 |
| 6 | neurally mediated syncope.mp. | 646 |
| 7 | Syncope, Vasovagal/ | 4859 |
| 8 | vasovagal syncope.mp. | 2241 |
| 9 | reflex syncope.mp. | 358 |
| 10 | situational syncope.mp. | 80 |
| 11 | neurogenic syncope.mp. | 51 |
| 12 | neurocardiogenic syncope.mp. | 738 |
| 13 | Postural Orthostatic Tachycardia Syndrome/ | 1434 |
| 14 | postural orthostatic tachycardia syndrome.mp. | 1524 |
| 15 | postural tachycardia syndrome.mp. | 677 |
| 16 | fainting.mp. | 1706 |
| 17 | faintness.mp. | 17424 |
| 18 | presyncope.mp. | 3375 |
| 19 | carotid sinus syncope.mp. | 94 |
| 20 | Carotid sinus syndrome.mp. | 669 |
| 21 | 1 or 2 or 3 or 4 or 5 or 6 or 7 or 8 or 9 or 10 or 11 or 12 or 13 or 14 or 15 or 16 or 17 or 18 or 19 or 20 | 45481 |
| 22 | Caffeine.mp. or CAFFEINE/ | 49778 |
| 23 | TEA/ or tea.mp. | 39857 |
| 24 | coffee.mp. or COFFEE/ | 18650 |
| 25 | 22 or 23 or 24 | 98545 |
| 26 | 21 and 25 | 393 |
| 27 | limit 26 to animals | 6 |
| 28 | limit 26 to "review" | 159 |
| 29 | 27 or 28 | 165 |
| 30 | 26 not 29 | 228 |

### PubMed (accessed 23/01/19)

260 results

(((((((((((((((((((((((orthostatic hypotension/) OR orthostatic hypotension.mp.) OR postural hypotension.mp.) OR Orthostatic Intolerance/) OR orthostatic intolerance.mp.) OR neurally mediated syncope.mp.) OR faintness/) OR Syncope, Vasovagal/) OR vasovagal syncope.mp.) OR reflex syncope.mp.) OR situational syncope.mp.) OR neurogenic syncope.mp.) OR neurocardiogenic syncope.mp.) OR Postural Orthostatic Tachycardia Syndrome/) OR postural orthostatic tachycardia syndrome.mp.) OR postural tachycardia syndrome.mp.) OR fainting.mp.) OR presyncope.mp.) OR carotid sinus syncope.mp.) OR Carotid sinus syndrome.mp.)))))) AND (((((((((((TEA/ or tea.mp)) OR (Caffeine/ OR Caffeine.mp)) OR (coffee.mp. or COFFEE/))))):

### Web of science (1970-2019 accessed 21/01/2019)

Couldn’t figure out how to take out animal studies or case reports

Kept autocorrecting ‘neurally’ to ‘neutrally’

| **Set** | **Results** | **Save History / Create Alert Open Saved History** |
| --- | --- | --- |
| # 6 | [**74**](http://apps.webofknowledge.com/summary.do?product=WOS&doc=1&qid=9&SID=E3NxoNZWMoNvtFt6L4T&search_mode=AdvancedSearch&update_back2search_link_param=yes) | (#4 not #5) *AND* **LANGUAGE:** (English)  *Indexes=SCI-EXPANDED, SSCI, A&HCI, CPCI-S, CPCI-SSH, ESCI Timespan=All years* |
| # 5 | [**7**](http://apps.webofknowledge.com/summary.do?product=WOS&doc=1&qid=8&SID=E3NxoNZWMoNvtFt6L4T&search_mode=AdvancedSearch&update_back2search_link_param=yes) | (#3) *AND* **LANGUAGE:** (English)  **Refined by:** **DOCUMENT TYPES:** ( REVIEW )  *Indexes=SCI-EXPANDED, SSCI, A&HCI, CPCI-S, CPCI-SSH, ESCI Timespan=All years* |
| # 4 | [**81**](http://apps.webofknowledge.com/summary.do?product=WOS&doc=1&qid=7&SID=E3NxoNZWMoNvtFt6L4T&search_mode=AdvancedSearch&update_back2search_link_param=yes) | (#3) *AND* **LANGUAGE:** (English)  *Indexes=SCI-EXPANDED, SSCI, A&HCI, CPCI-S, CPCI-SSH, ESCI Timespan=All years* |
| # 3 | [**86**](http://apps.webofknowledge.com/summary.do?product=WOS&doc=1&qid=6&SID=E3NxoNZWMoNvtFt6L4T&search_mode=CombineSearches&update_back2search_link_param=yes) | #2 AND #1  *Indexes=SCI-EXPANDED, SSCI, A&HCI, CPCI-S, CPCI-SSH, ESCI Timespan=All years* |
| # 2 | [**112,245**](http://apps.webofknowledge.com/summary.do?product=WOS&doc=1&qid=5&SID=E3NxoNZWMoNvtFt6L4T&search_mode=AdvancedSearch&update_back2search_link_param=yes) | TS=(Caffeine) OR TS=(Coffee) OR TS=(Tea)  *Indexes=SCI-EXPANDED, SSCI, A&HCI, CPCI-S, CPCI-SSH, ESCI Timespan=All years* |
| # 1 | [**34,672**](http://apps.webofknowledge.com/summary.do?product=WOS&doc=1&qid=4&SID=E3NxoNZWMoNvtFt6L4T&search_mode=AdvancedSearch&update_back2search_link_param=yes) | TS=(orthostatic hypotension) OR TS=(postural hypotension) OR TS=(orthostatic intolerance) OR TS=(neural mediated syncope) OR TS=(faintness) OR TS=(vasovagal syncope) OR TS=(reflex syncope) OR TS=(situational syncope) OR TS=(neurogenic syncope) OR TS=(neurocardiogenic syncope) OR TS=(postural orthostatic tachycardia syndrome) OR TS=(postural tachycardia syndrome) OR TS=(fainting) OR TS=(Presyncope) OR TS=(carotid sinus syndrome) OR TS=(Carotid sinus syncope)  *Indexes=SCI-EXPANDED, SSCI, A&HCI, CPCI-S, CPCI-SSH, ESCI Timespan=All years* |

### Scopus accessed 21/01/2019

( ( TITLE-ABS-KEY ( orthostatic AND hypotension ) ) OR ( TITLE-ABS-KEY ( orthostatic AND intolerance ) ) OR ( TITLE-ABS-KEY ( postural AND hypotension ) ) OR ( TITLE-ABS-KEY ( neurally AND mediated AND syncope ) ) OR ( TITLE-ABS-KEY ( faintness ) ) OR ( TITLE-ABS-KEY ( vasovagal AND syncope ) ) OR ( TITLE-ABS-KEY ( reflex AND syncope ) ) OR ( TITLE-ABS-KEY ( situational AND syncope ) ) OR ( TITLE-ABS-KEY ( neurogenic AND syncope ) ) OR ( TITLE-ABS-KEY ( neurocardiogenic AND syncope ) ) OR ( TITLE-ABS-KEY ( postural AND orthostatic AND tachycardia AND syndrome ) ) OR ( TITLE-ABS-KEY ( postural AND tachycardia AND syndrome ) ) OR ( TITLE-ABS-KEY ( fainting ) ) OR ( TITLE-ABS-KEY ( presyncope ) ) OR ( TITLE-ABS-KEY ( carotid AND sinus AND syncope ) ) OR ( TITLE-ABS-KEY ( carotid AND sinus AND syndrome ) ) ) AND ( ( TITLE-ABS-KEY ( caffeine ) ) OR ( TITLE-ABS-KEY ( tea ) ) OR ( TITLE-ABS-KEY ( coffee ) ) ) AND ( LIMIT-TO ( LANGUAGE , "English" ) ) AND ( EXCLUDE ( DOCTYPE , "re" ) )

Results = 221

### Open Grey (database for information on Grey literature in Europe) accessed 21/01/2019

orthostatic hypotension OR postural hypotension OR orthostatic intolerance OR neurally mediated syncope OR faintness OR vasovagal syncope OR reflex syncope OR situational syncope OR neurogenic syncope OR postural orthostatic tachycardia syndrome OR postural tachycardia syndrome OR fainting OR Presyncope OR carotid sinus syndrome OR carotid sinus syncope =

only 39 results, 0 if combined with caffeine search terms (?due to search syntax)

<http://apps.who.int/trialsearch/AdvSearch.aspx>

### Proquest 1970-2019 accessed 23/01/2019

(MESH(hypotension, orthostatic) OR MESH(orthostatic intolerance) OR MESH(Posture) OR MESH(faintness) OR MESH(syncope, vasovagal) OR MESH(reflex syncope) OR MESH(situational syncope) OR MESH(neurogenic syncope) OR MESH(neurocardiogenic syncope) OR MESH(postural orthostatic tachycardia syndrome) OR MESH(postural tachycardia syndrome) OR MESH(fainting) OR MESH(presyncope) OR MESH(carotid sinus syncope)) AND (MESH(Caffeine) OR MESH(Tea) OR MESH(Coffee)) AND YR(1970-2019)

58 results, but reviews and animal studies not excluded.

### ISRCTN registry accessed 23/01/2019

(("orthostatic hypotension") OR ("orthostatic intolerance") OR ("postural hypotension") OR ("neurally mediated syncope") OR (“vasovagal syncope”) OR (“reflex syncope”) OR (“situational syncope”) OR (“neurogenic syncope”) OR (“neurocardiogenic syncope”) OR (“postural orthostatic tachycardia syndrome”) OR (“postural tachycardia syndrome”)) AND ((“caffeine”) OR (“tea”) OR (“coffee))

5 results

# Database searches January 2021

## Medline January 2019 to week 2 January 2021

| **#** | **Searches** | **Results** |
| --- | --- | --- |
| 1 | Hypotension, Orthostatic/ | 5769 |
| 2 | orthostatic hypotension.mp. | 4875 |
| 3 | postural hypotension.mp. | 1371 |
| 4 | Orthostatic Intolerance/ | 330 |
| 5 | orthostatic intolerance.mp. | 1261 |
| 6 | neurally mediated syncope.mp. | 437 |
| 7 | Syncope, Vasovagal/ | 1965 |
| 8 | vasovagal syncope.mp. | 1362 |
| 9 | reflex syncope.mp. | 200 |
| 10 | situational syncope.mp. | 35 |
| 11 | neurogenic syncope.mp. | 36 |
| 12 | neurocardiogenic syncope.mp. | 457 |
| 13 | Postural Orthostatic Tachycardia Syndrome/ | 504 |
| 14 | postural orthostatic tachycardia syndrome.mp. | 634 |
| 15 | postural tachycardia syndrome.mp. | 401 |
| 16 | fainting.mp. | 1077 |
| 17 | presyncope.mp. | 990 |
| 18 | carotid sinus syncope.mp. | 83 |
| 19 | Carotid sinus syndrome.mp. | 372 |
| 20 | 1 or 2 or 3 or 4 or 5 or 6 or 7 or 8 or 9 or 10 or 11 or 12 or 13 or 14 or 15 or 16 or 17 or 18 or 19 | 14320 |
| 21 | Caffeine.mp. or CAFFEINE/ | 32272 |
| 22 | TEA/ or tea.mp. | 28256 |
| 23 | coffee.mp. or COFFEE/ | 13696 |
| 24 | 21 or 22 or 23 | 67537 |
| 25 | 20 and 24 | 39 |
| 26 | limit 25 to animals | 4 |
| 27 | limit 25 to "review" | 8 |
| 28 | 26 or 27 | 12 |
| 29 | 25 not 28 | 26 |
| 30 | Limit 29 to yr = “2019 – 2021” | 1 |

1 extra article identified, excluded in primary screening.

## Embase (1974 to 2021 January 22)

| **#** | **Searches** | **Results** |
| --- | --- | --- |
| 1 | Hypotension, Orthostatic/ | 14172 |
| 2 | orthostatic hypotension.mp. | 22943 |
| 3 | postural hypotension.mp. | 1987 |
| 4 | Orthostatic Intolerance/ | 1191 |
| 5 | orthostatic intolerance.mp. | 2449 |
| 6 | neurally mediated syncope.mp. | 695 |
| 7 | Syncope, Vasovagal/ | 9280 |
| 8 | vasovagal syncope.mp. | 2541 |
| 9 | reflex syncope.mp. | 436 |
| 10 | situational syncope.mp. | 95 |
| 11 | neurogenic syncope.mp. | 51 |
| 12 | neurocardiogenic syncope.mp. | 785 |
| 13 | Postural Orthostatic Tachycardia Syndrome/ | 1910 |
| 14 | postural orthostatic tachycardia syndrome.mp. | 2014 |
| 15 | postural tachycardia syndrome.mp. | 836 |
| 16 | fainting.mp. | 1896 |
| 17 | faintness.mp. | 23095 |
| 18 | presyncope.mp. | 4145 |
| 19 | carotid sinus syncope.mp. | 97 |
| 20 | Carotid sinus syndrome.mp. | 719 |
| 21 | 1 or 2 or 3 or 4 or 5 or 6 or 7 or 8 or 9 or 10 or 11 or 12 or 13 or 14 or 15 or 16 or 17 or 18 or 19 or 20 | 45481 |
| 22 | Caffeine.mp. or CAFFEINE/ | 54612 |
| 23 | TEA/ or tea.mp. | 46726 |
| 24 | coffee.mp. or COFFEE/ | 22328 |
| 25 | 22 or 23 or 24 | 112372 |
| 26 | 21 and 25 | 393 |
| 27 | limit 26 to animals | 6 |
| 28 | limit 26 to "review" | 159 |
| 29 | 27 or 28 | 165 |
| 30 | 26 not 29 | 228 |
| 31 | limit 30 to yr="2019 - 2021" | 37 |

37 extra articles identified, all excluded in primary screening.

## PubMed (accessed 22/01/21)

(((((((((((((((((((((((orthostatic hypotension/) OR orthostatic hypotension.mp.) OR postural hypotension.mp.) OR Orthostatic Intolerance/) OR orthostatic intolerance.mp.) OR neurally mediated syncope.mp.) OR faintness/) OR Syncope, Vasovagal/) OR vasovagal syncope.mp.) OR reflex syncope.mp.) OR situational syncope.mp.) OR neurogenic syncope.mp.) OR neurocardiogenic syncope.mp.) OR Postural Orthostatic Tachycardia Syndrome/) OR postural orthostatic tachycardia syndrome.mp.) OR postural tachycardia syndrome.mp.) OR fainting.mp.) OR presyncope.mp.) OR carotid sinus syncope.mp.) OR Carotid sinus syndrome.mp.)))))) AND (((((((((((TEA/ or tea.mp)) OR (Caffeine/ OR Caffeine.mp)) OR (coffee.mp. or COFFEE/))))):

New articles from 21/01/2019 – 22/01/2021: 31 – all excluded in primary screening.

## Web of science (1970-2021 accessed 22/01/2021)

7 extra articles identified, all excluded in primary screening

| **Set** | **Results** | **Save History / Create Alert Open Saved History** |
| --- | --- | --- |
| # 6 | [**81**](http://apps.webofknowledge.com/summary.do?product=WOS&doc=1&qid=9&SID=E3NxoNZWMoNvtFt6L4T&search_mode=AdvancedSearch&update_back2search_link_param=yes) | (#4 not #5) *AND* **LANGUAGE:** (English)  *Indexes=SCI-EXPANDED, SSCI, A&HCI, CPCI-S, CPCI-SSH, ESCI Timespan=All years* |
| # 5 | [**10**](http://apps.webofknowledge.com/summary.do?product=WOS&doc=1&qid=8&SID=E3NxoNZWMoNvtFt6L4T&search_mode=AdvancedSearch&update_back2search_link_param=yes) | (#3) *AND* **LANGUAGE:** (English)  **Refined by:** **DOCUMENT TYPES:** ( REVIEW )  *Indexes=SCI-EXPANDED, SSCI, A&HCI, CPCI-S, CPCI-SSH, ESCI Timespan=All years* |
| # 4 | [**91**](http://apps.webofknowledge.com/summary.do?product=WOS&doc=1&qid=7&SID=E3NxoNZWMoNvtFt6L4T&search_mode=AdvancedSearch&update_back2search_link_param=yes) | (#3) *AND* **LANGUAGE:** (English)  *Indexes=SCI-EXPANDED, SSCI, A&HCI, CPCI-S, CPCI-SSH, ESCI Timespan=All years* |
| # 3 | [**96**](http://apps.webofknowledge.com/summary.do?product=WOS&doc=1&qid=6&SID=E3NxoNZWMoNvtFt6L4T&search_mode=CombineSearches&update_back2search_link_param=yes) | #2 AND #1  *Indexes=SCI-EXPANDED, SSCI, A&HCI, CPCI-S, CPCI-SSH, ESCI Timespan=All years* |
| # 2 | [**131,165**](http://apps.webofknowledge.com/summary.do?product=WOS&doc=1&qid=5&SID=E3NxoNZWMoNvtFt6L4T&search_mode=AdvancedSearch&update_back2search_link_param=yes) | TS=(Caffeine) OR TS=(Coffee) OR TS=(Tea)  *Indexes=SCI-EXPANDED, SSCI, A&HCI, CPCI-S, CPCI-SSH, ESCI Timespan=All years* |
| # 1 | [**34,672**](http://apps.webofknowledge.com/summary.do?product=WOS&doc=1&qid=4&SID=E3NxoNZWMoNvtFt6L4T&search_mode=AdvancedSearch&update_back2search_link_param=yes) | TS=(orthostatic hypotension) OR TS=(postural hypotension) OR TS=(orthostatic intolerance) OR TS=(neural mediated syncope) OR TS=(faintness) OR TS=(vasovagal syncope) OR TS=(reflex syncope) OR TS=(situational syncope) OR TS=(neurogenic syncope) OR TS=(neurocardiogenic syncope) OR TS=(postural orthostatic tachycardia syndrome) OR TS=(postural tachycardia syndrome) OR TS=(fainting) OR TS=(Presyncope) OR TS=(carotid sinus syndrome) OR TS=(Carotid sinus syncope)  *Indexes=SCI-EXPANDED, SSCI, A&HCI, CPCI-S, CPCI-SSH, ESCI Timespan=All years* |

## Scopus accessed 22/01/2021

( ( TITLE-ABS-KEY ( orthostatic AND hypotension ) ) OR ( TITLE-ABS-KEY ( orthostatic AND intolerance ) ) OR ( TITLE-ABS-KEY ( postural AND hypotension ) ) OR ( TITLE-ABS-KEY ( neurally AND mediated AND syncope ) ) OR ( TITLE-ABS-KEY ( faintness ) ) OR ( TITLE-ABS-KEY ( vasovagal AND syncope ) ) OR ( TITLE-ABS-KEY ( reflex AND syncope ) ) OR ( TITLE-ABS-KEY ( situational AND syncope ) ) OR ( TITLE-ABS-KEY ( neurogenic AND syncope ) ) OR ( TITLE-ABS-KEY ( neurocardiogenic AND syncope ) ) OR ( TITLE-ABS-KEY ( postural AND orthostatic AND tachycardia AND syndrome ) ) OR ( TITLE-ABS-KEY ( postural AND tachycardia AND syndrome ) ) OR ( TITLE-ABS-KEY ( fainting ) ) OR ( TITLE-ABS-KEY ( presyncope ) ) OR ( TITLE-ABS-KEY ( carotid AND sinus AND syncope ) ) OR ( TITLE-ABS-KEY ( carotid AND sinus AND syndrome ) ) ) AND ( ( TITLE-ABS-KEY ( caffeine ) ) OR ( TITLE-ABS-KEY ( tea ) ) OR ( TITLE-ABS-KEY ( coffee ) ) ) AND ( LIMIT-TO ( LANGUAGE , "English" ) ) AND ( EXCLUDE ( DOCTYPE , "re" ) )

Results = 245 (24 additional articles identified, all excluded in primary screening)

## Open Grey (database for information on Grey literature in Europe) accessed 22/01/2021

orthostatic hypotension OR postural hypotension OR orthostatic intolerance OR neurally mediated syncope OR faintness OR vasovagal syncope OR reflex syncope OR situational syncope OR neurogenic syncope OR postural orthostatic tachycardia syndrome OR postural tachycardia syndrome OR fainting OR Presyncope OR carotid sinus syndrome OR carotid sinus syncope =

39 results, no additional results from 2019 search

## Proquest 1970-2019 accessed 22/01/2021

(MESH(hypotension, orthostatic) OR MESH(orthostatic intolerance) OR MESH(Posture) OR MESH(faintness) OR MESH(syncope, vasovagal) OR MESH(reflex syncope) OR MESH(situational syncope) OR MESH(neurogenic syncope) OR MESH(neurocardiogenic syncope) OR MESH(postural orthostatic tachycardia syndrome) OR MESH(postural tachycardia syndrome) OR MESH(fainting) OR MESH(presyncope) OR MESH(carotid sinus syncope)) AND (MESH(Caffeine) OR MESH(Tea) OR MESH(Coffee)) AND YR(1970-2019)

59 results, 1 additional result from 2019 search, excluded in primary screening.

## ISRCTN registry accessed 22/01/2021

(("orthostatic hypotension") OR ("orthostatic intolerance") OR ("postural hypotension") OR ("neurally mediated syncope") OR (“vasovagal syncope”) OR (“reflex syncope”) OR (“situational syncope”) OR (“neurogenic syncope”) OR (“neurocardiogenic syncope”) OR (“postural orthostatic tachycardia syndrome”) OR (“postural tachycardia syndrome”)) AND ((“caffeine”) OR (“tea”) OR (“coffee))

8 results, 3 additional results since 2019, all excluded in primary screening

<http://apps.who.int/trialsearch/AdvSearch.aspx>
